# Supplementary material for: Metagenomic binning with assembly graph embeddings
Source: Bioinformatics. 2022 Aug 16;38(19):4481–7. doi: 10.1093/bioinformatics/btac557 (PMC9525014; doi:10.1093/bioinformatics/btac557)
Supplement: btac557_supplementary_data [file btac557_supplementary_data.zip › amber outputs.zip!/amber outputs/strong100/semibin_results/genome/semibin/purity_vs_bin_size_SAMPLEID.pdf]

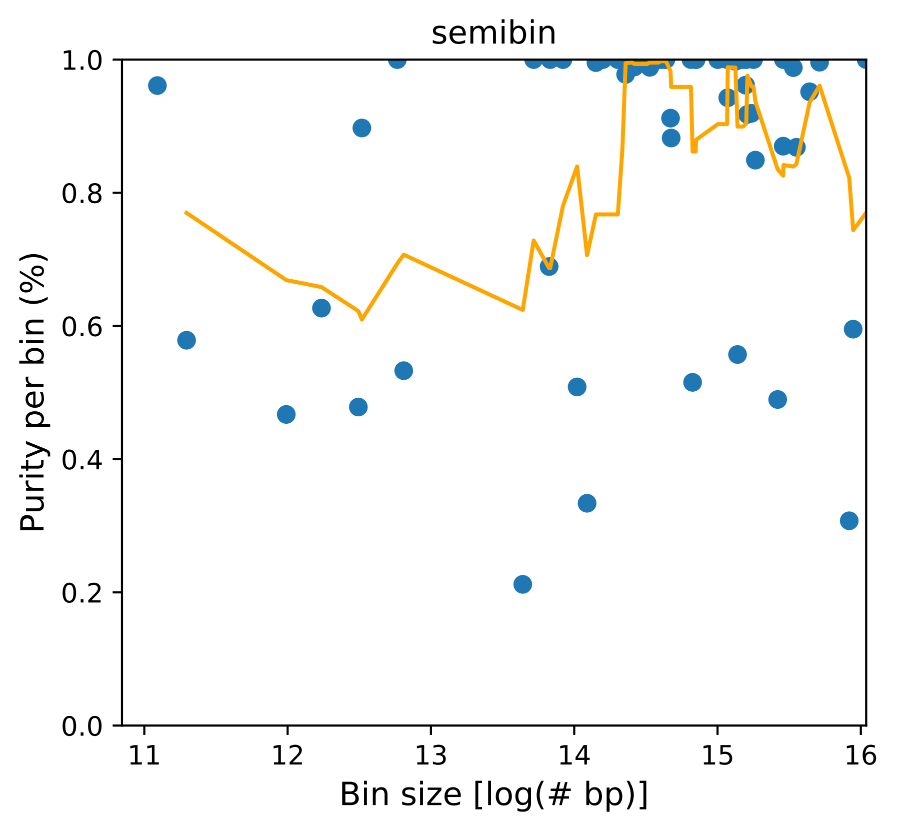
semibin
Purity per bin (%)
Bin size [log(# bp)]
0.0
0.2
0.4
0.6
0.8
1.0
11
12
13
14
15
16
